# Parental cancer as a risk factor for nine common childhood malignancies

**DOI:** 10.1054/bjoc.2000.1629

**Published:** 2001-04

**Authors:** K Hemminki, P Mutanen

**Affiliations:** Department of Biosciences at Novum, Karolinska Institute, 141 57 Huddinge, Sweden

**Keywords:** brain cancer, leukaemia, lymphoma, Wilms tumour, retinoblastoma endocrine tumours

## Abstract

The nationwide Swedish Family-Cancer Database was used to analyse childhood tumours among 8158 offspring by parental cancers. The results showed 2-fold familial increases for nervous system cancers and lymphomas, a 6.4-fold increase for endocrine tumours, a 60-fold increased risk for retinoblastomas but no excess risk for leukaemia and Wilms tumour. © 2001 Cancer Research Campaign http://www.bjcancer.com


*British Journal of Cancer* (2001) **84**(7), 990–993
© 2001 Cancer Research Campaign
doi: 10.1054/ bjoc.2000.1629, available online at http://www.idealibrary.com on IDEAL®

http://www.bjcancer.com

Short Communication

### Parental cancer as a risk factor for nine common childhood malignancies

**K Hemminki and P Mutanen***

Department of Biosciences at Novum, Karolinska Institute, 141 57 Huddinge, Sweden.

**Summary** The nationwide Swedish Family-Cancer Database was used to analyse childhood tumours among 8158 offspring by parental cancers. The results showed 2-fold familial increases for nervous system cancers and lymphomas, a 6.4-fold increase for endocrine tumours, a 60-fold increased risk for retinoblastomas but no excess risk for leukaemia and Wilms tumour. © 2001 Cancer Research Campaign http://www.bjcancer.com

**Keywords**: brain cancer; leukaemia; lymphoma; Wilms tumour; retinoblastoma endocrine tumours

Nervous system tumours and acute leukaemia are the most common malignancies in children under 15 years of age, followed by lymphomas, Wilms tumours and retinoblastomas (Draper et al, 1994; IARC, 1998; Linet et al, 1999). Over the past decades there has been a modest increase in the reported childhood malignancies, particularly of nervous system tumours and leukaemia, in many countries, but at least part of the increase can be ascribed to improvements in diagnostics and reporting (Draper et al, 1994; IARC, 1998; Hemminki et al, 1999; Linet et al, 1999). Despite extensive studies, the causes of childhood cancer have remained largely unknown. Ionizing radiation and genetic predisposition are established risk factors of certain types of childhood malignancies (Bondy et al, 1991; Draper et al, 1994, 1996, 1997; Zahm and Devessa, 1995; Little, 1999; Kleihues and Cavenee, 2000). Parental age, perinatal factors and infections in childhood may play a role in brain cancer and leukaemia (Shaw et al, 1984; Emerson et al, 1991; Kaye et al, 1991; Savitz and Ananth, 1994; Cnattingius et al, 1995; Kinlen et al, 1995; Dockerty et al, 1999; Hemminki et al, 1999).

We analyse the risk for nervous system cancers, leukaemias, lymphomas, Wilms tumours, retinoblastomas and endocrine gland tumours by parental cancers at any age using the nationwide Swedish Family-Cancer Database (Hemminki et al, 1998; Hemminki and Vaittinen, 1999; Hemminki and Dong, 2000). The Database offers unique possibilities for reliable estimation of familial risks, because the data on family relationships and cancers were obtained from registered sources of practically complete coverage (Hemminki and Vaittinen, 1998a; Hemminki et al, 1998). We use the 1999 update of the nationwide Swedish Family-Cancer Database, covering 9.6 million individuals, more than the country's population of 8.8 million.

The Swedish Family-Cancer Database, updated in 1999, includes persons, 'offspring' born in Sweden after 1934 with their biological parents, totalling over 9.6 million individuals (Hemminki and Vaittinen, 1998b; Hemminki et al, 1998). Cancers were retrieved from the nationwide Swedish Cancer Registry from years 1958 to 1996. A 4-digit diagnostic code according to the 7th revision of the International Classification of Diseases (ICD-7) was used with 3 or 4 digits; for retinoblastoma and Wilms tumour, pathology codes were also used for classification.

The analysis was carried out on all types of malignancies diagnosed before age 15 between years 1958 and 1996. Explanatory variables included in the statistical analysis were parental cancer status, socio-economic status (SES, 4-category variable: agriculture, professional, worker, other) and area of living (county, five-category variable: Stockholm area, the largest city; Gothenburg-Malmö area, two largest cities in south of Sweden; Götaland, Svealand and Norrland, three geographic regions, from south to north, respectively). The SES and area variables were extracted from the national census 1960 of Statistics Sweden. All analysis also included two variables: age at diagnosis (five-year categories) and year of birth (birth cohort, three categories: 1941–1950, 1951–1960, 1961–1996). Standardized incidence ratios (SIRs) were calculated as the ratio of observed (O) to expected (E) number of cases. The expected numbers were calculated from 5-year-age- and tumour type-specific standard incidence rates (Esteve et al, 1994). Confidence intervals (95% CI) were calculated assuming a Poisson distribution (Esteve et al, 1994).

The Family-Cancer Database covered years 1958 to 1996 from the Swedish Cancer Registry and included 8158 cases of childhood tumours, diagnosed before age 15 (Table 1). The most common types were nervous system tumours (2486), leukaemias (2397) and lymphomas (807).

SIRs for the 9 most common childhood tumours by parental cancer are shown in Table 2. When neither parent had cancer the SIRs ranged from 0.90 to 1.01. When either father or mother had any cancer the SIRs ranged from 0.92 or 0.93 (leukaemia and Wilms tumour, respectively) to 1.22 for lymphoma and 1.44 for retinoblastoma, which were significant. When both parents had any cancer, the SIR for lymphoma was 1.93 and that for endocrine gland tumour 4.84, both statistically significant. In these families, the parental cancer types were heterogeneous and did not reveal a special pattern.

*Received 4 October 2000*
*Revised 17 November 2000*
*Accepted 17 November 2000*

*Correspondence to:* K Hemminki

* On leave from the Finnish Institute of Occupational Health.

**Table 1** Number of childhood (<15 years) cancers during 1958–1996 according to cancer type and gender

| Cancer type | Male | % | Female | % | All | % |
|---|---|---|---|---|---|---|
| Nervous system | 1317 | 29.9 | 1169 | 31.1 | 2486 | 30.5 |
| Leukaemia | 1298 | 29.5 | 1099 | 29.3 | 2397 | 29.4 |
| Lymphoma | 564 | 12.8 | 243 | 6.5 | 807 | 9.9 |
| Wilms tumour | 239 | 5.4 | 264 | 7.0 | 503 | 6.2 |
| Bone | 178 | 4.0 | 172 | 4.6 | 350 | 4.3 |
| Connective tissues | 165 | 3.7 | 156 | 4.2 | 321 | 3.9 |
| Retinoblastoma | 141 | 3.2 | 128 | 3.4 | 269 | 3.3 |
| Endocrine | 104 | 2.4 | 77 | 2.1 | 181 | 2.2 |
| Thyroid | 16 | 0.4 | 61 | 1.6 | 77 | 0.9 |
| Other | 380 | 8.6 | 387 | 10.3 | 767 | 9.4 |
| All cancers | 4402 | 100.0 | 3756 | 100.0 | 8158 | 100.0 |

**Table 2** Childhood cancer by any parental cancer

| Offspring cancer | Parental cancer | | | | | | | | | | | |
|---|---|---|---|---|---|---|---|---|---|---|---|---|
| | No parental cancer | | | | Any parental cancer | | | | Any cancer in both parents | | | |
| | O | SIR | 95% CI | | O | SIR | 95% CI | | O | SIR | 95% CI | |
| Nervous system | 1954 | 1.00 | 0.96 | 1.04 | 256 | 1.02 | 0.90 | 1.15 | 20 | 1.10 | 0.67 | 1.70 |
| Leukaemia | 1906 | 1.01 | 0.96 | 1.05 | 211 | 0.92 | 0.80 | 1.06 | 21 | 1.35 | 0.83 | 2.06 |
| Lymphoma | 612 | 0.96 | 0.89 | 1.04 | 106 | 1.22 | 1.00 | 1.47 | 13 | 1.93 | 1.03 | 3.29 |
| Wilms tumour | 394 | 1.01 | 0.91 | 1.11 | 41 | 0.93 | 0.67 | 1.27 | 3 | 1.10 | 0.23 | 3.21 |
| Bone | 268 | 0.96 | 0.85 | 1.08 | 56 | 1.16 | 0.87 | 1.50 | 7 | 1.68 | 0.68 | 3.46 |
| Connective tissues | 243 | 0.98 | 0.86 | 1.11 | 36 | 1.20 | 0.84 | 1.66 | 1 | 0.47 | 0.01 | 2.62 |
| Retinoblastoma | 197 | 0.95 | 0.82 | 1.09 | 34 | 1.45 | 1.00 | 2.02 | 2 | 1.42 | 0.17 | 5.14 |
| Endocrine | 130 | 0.96 | 0.80 | 1.14 | 17 | 1.09 | 0.63 | 1.74 | 5 | 4.84 | 1.57 | 11.29 |
| Thyroid | 52 | 0.90 | 0.67 | 1.18 | 18 | 1.48 | 0.88 | 2.34 | 1 | 0.88 | 0.02 | 4.88 |

**Table 3** Childhood cancer by parental cancer type

| Offspring cancer | Parental cancer type | | | | | | | |
|---|---|---|---|---|---|---|---|---|
| | Nervous system | | | | Endocrine | | | |
| | O | SIR | 95% CI | | O | SIR | 95% CI | |
| Nervous system | 28 | 2.03 | 1.35 | 2.93 | 4 | 0.54 | 0.15 | 1.38 |
| Leukaemia | 12 | 0.93 | 0.48 | 1.63 | 6 | 0.88 | 0.32 | 1.91 |
| Lymphoma | 1 | 0.21 | 0.01 | 1.19 | 2 | 0.79 | 0.10 | 2.84 |
| Wilms tumour | 5 | 1.97 | 0.64 | 4.60 | 0 | – | – | – |
| Bone | 1 | 0.41 | 0.01 | 2.27 | 1 | 0.73 | 0.02 | 4.07 |
| Connective tissues | 1 | 0.60 | 0.02 | 3.32 | 2 | 2.24 | 0.27 | 8.08 |
| Retinoblastoma | 2 | 1.47 | 0.18 | 5.31 | 0 | – | – | – |
| Endocrine | 5 | 5.67 | 1.84 | 13.22 | 3 | 6.37 | 1.31 | 18.60 |
| Thyroid | 0 | – | – | – | 3 | 8.95 | 1.85 | 26.16 |

SIRs of childhood cancers by parental nervous system and endocrine gland tumour are shown in Table 3. For nervous system cancer, 28 child–parent pairs were identified, giving a SIR of 2.03. Most tumours (25/28) in offspring were located in the brain. In one pair, even the parental tumour (ependymoma) was diagnosed in childhood. In another pair, both the offspring and the parent had cerebral haemangioblastoma. In 7 pairs, both the offspring and the parent presented with cerebral astrocytoma; in 5 pairs, offspring presented with cerebral astrocytoma, the parents with a cerebral meningioma; in three pairs, offspring presented with cerebral astrocytoma, the parent with peripheral or spinal neurinoma. The remaining pairs were mixed single histologies. Offspring endocrine gland tumours were associated with parental nervous system tumours, giving a SIR of 5.67. Of these, three were adrenal ade1nocarcinomas (offspring) – cerebral astrocytomas (parent), one pair was adrenal ganglioneurinoma – brain neurinoma, and one pair was pancreatic adenoma–brain meningioma.

Parental endocrine gland tumours were associated with thyroid and other endocrine gland tumours in offspring. The thyroid (offspring)–endocrine (parent) pairs were one medullary thyroid cancer–adrenal paraganglioma, one non-medullary thyroid cancer–adrenal adenocarcinoma, and one undifferentiated thyroid cancer–adrenal paraganglioma.

Associations of childhood cancers with parental leukaemia, lymphomas, kidney cancers and retinoblastomas are shown in Table 4. Parental leukaemia and kidney cancer associated with no

© 2001 Cancer Research Campaign

**Table 4** Childhood cancer by the parental cancer type

| Offspring cancer | Leukaemia | | | | Lymphoma | | | | Kidney | | | | Retinoblastoma | | | |
|---|---|---|---|---|---|---|---|---|---|---|---|---|---|---|---|---|
| | O | SIR | 95% CI | | O | SIR | 95% CI | | O | SIR | 95% CI | | O | SIR | 95% CI | |
| Nervous system | 7 | 0.91 | 0.37 | 1.88 | 11 | 0.91 | 0.46 | 1.63 | 12 | 1.31 | 0.68 | 2.29 | 1 | 0.94 | 0.02 | 5.23 |
| Leukaemia | 6 | 0.86 | 0.31 | 1.86 | 14 | 1.26 | 0.69 | 2.12 | 8 | 0.97 | 0.42 | 1.91 | 1 | 1.01 | 0.03 | 5.65 |
| Lymphoma | 2 | 0.75 | 0.09 | 2.70 | 9 | 2.19 | 1.00 | 4.16 | 4 | 1.25 | 0.34 | 3.20 | 1 | 2.79 | 0.07 | 15.55 |
| Wilms | 3 | 2.24 | 0.46 | 6.55 | 1 | 0.46 | 0.01 | 2.57 | 0 | – | – | – | 0 | – | – | – |
| Bone | 2 | 1.34 | 0.16 | 4.86 | 2 | 0.90 | 0.11 | 3.24 | 3 | 1.60 | 0.33 | 4.68 | 1 | 5.10 | 0.13 | 28.42 |
| Connective tissues | 1 | 1.06 | 0.03 | 5.90 | 1 | 0.69 | 0.02 | 3.82 | 1 | 0.91 | 0.02 | 5.10 | 0 | – | – | – |
| Retinoblastoma | 0 | – | – | – | 3 | 2.58 | 0.53 | 7.53 | 0 | – | – | – | 6 | 57.97 | 21.27 | 126.17 |
| Endocrine | 2 | 4.32 | 0.52 | 15.59 | 2 | 2.63 | 0.32 | 9.50 | 0 | – | – | – | 0 | – | – | – |
| Thyroid | 0 | – | – | – | 0 | – | – | – | 0 | – | – | – | 0 | – | – | – |

(Parental cancer type spans Leukaemia, Lymphoma, Kidney and Retinoblastoma.)

childhood cancer. Concordant lymphomas showed a SIR of 2.19 of borderline significance. Concordant retinoblastoma showed a SIR of 57.97. All the 6 retinoblastoma pairs were diagnosed before age 4, even among parents.

No data are shown for other childhood cancers by other parental cancer because at most a single offspring–parent pair with tumours was identified.

Among the present childhood malignancies, retinoblastoma and Wilms tumour are well-defined clinical entities. The main cause of retinoblastoma is mutation in the *RB1* gene. About 40% of retinoblastomas are hereditary and a quarter of these are new germinal mutations (Lindor et al, 1998). Practically all the hereditary cases are diagnosed in the first years of life, in agreement with our data. The SIR for retinoblastoma, 57.97, was the highest found in the present study. *RB1* mutations predispose also to other tumours, such as osteosarcoma. In our material there were too few cases to confirm the relationship. For Wilms tumour, at least three gene loci, *WT1*, *WT2* and *WT3* have been reported (Lindor et al, 1998). Less that 1% of Wilms tumour is hereditary and we found no indication of increase in childhood Wilms tumours by parental cancer.

Childhood brain cancer is encompassed in many rare cancer syndromes of high risk (Draper et al, 1996), but hereditary effects have been ascribed only to some 2–4% of brain cancers (Bondy et al, 1991; Hemminki et al, 2000c; Narod et al, 1991). In population-based studies, the familial risk of brain tumours was 2.5, and in offspring of survivors of childhood brain tumours it was 2.0 (Sankila et al, 1998; Hemminki et al, 2000c). However, a study on cancer in parents of childhood cancer probands found no increase in the risk of nervous system cancer between the two generations (Olsen et al, 1995). In a previous study on childhood brain cancer we noted a risk of 10.26 in childhood astrocytoma when a parent had meningioma (Hemminki et al, 2000c). Among adult brain tumours, we have reported a familial risk of 1.70 and another study from Sweden reported an aggregation of adult astrocytomas (Malmer et al, 1999; Hemminki et al, 2000a). Our present findings were in line with the above reports, because 25 of the 28 familial nervous system cancers were brain tumours. Li-Fraumeni syndrome features the occurrence of diverse gliomas (Sedlacek et al, 1998), and it is possible that some of the astrocytoma aggregates were due to this syndrome. The astrocytoma-meningioma combination may be due to neurofibromatosis, particularly of type 2 (Huson, 1998). One child–parent pair presented with brain haemangioblastoma, pathognomonic of von Hippel-Lindau syndrome (Lindor et al, 1998; Hemminki et al, 2000b).

Endocrine gland tumours manifest in several known cancer syndromes. The aggregation of endocrine gland tumours, particularly parathyroid adenomas, among children and parents suggests involvement of multiple endocrine neoplasia 1. In one pair, adrenal paraganglioma in offspring was associated with parental medullary thyroid tumour, signalling the presence of multiple endocrine neoplasia 2 (Hemminki and Dong, 2000). Offspring endocrine tumours were associated with parental nervous system tumours, but not vice versa. The aggregation of nervous system and endocrine tumours is common to neurofibromatosis 1 (Lindor et al, 1998).

Among haematopoietic malignancies, only lymphoma showed familial aggregation. The SIR of 2.19 in children was higher than the risk (1.5) previously found for the adult population from the Database (Hemminki et al, 1998). On the other hand, leukaemia showed no familial effect although such is present in the adult population, showing a relative risk of 1.8 (Hemminki et al, 1998). The common childhood leukaemia, acute lymphocytic leukaemia is uncommon among adults, perhaps explaining the absence of a familial effect.

The present data showed about 2-fold familial effects in nervous system tumours and lymphomas, a 6.4-fold risk in endocrine tumours and close to a 60-fold risk in retinoblastoma. No familial effect was observed in leukaemia and Wilms tumour. Aggregation of nervous system and endocrine gland tumours was noted.

### ACKNOWLEDGEMENTS

Supported by the Childhood Cancer Fund.

### REFERENCES

Bondy ML, Lustbader ED, Buffler PA, Schull WJ, Hardy RJ and Strong LC (1991) Genetic epidemiology of childhood brain tumors. *Genet Epidemiol* **8**: 253–267

Cnattingius S, Zack M, Ekbom A, Gunnarskog J, Kreuger A, Linet M and Adami H (1995) Prenatal and neonatal risk factora for childhood lymphatic leukemia. *J Natl Cancer Inst* **87**: 908–914

Dockerty J, Skegg D, Elwood J, Herbison G, Becroft D and Lewis M (1999) Infections, vaccinations, and the risk of childhood leukaemia. *Br J Cancer* **80**: 1483–1489

Draper G, Kroll M and Stiller C (1994) Childhood cancer. *Cancer Surveys: Trend in Cancer Incidence and Mortality* **19**/**20**: 493–517

Draper G, Sanders B, Lennox E and Brownbill P (1996) Patterns of childhood cancer among sibs. *Br J Cancer* **74**: 152–158

Draper G, Heaf M and Kinner Wilson L (1997) Occurence of childhood cancers among sibs and estimation of familial risks. *J Med Genet* **14**: 81–90

 *© 2001 Cancer Research Campaign*

Emerson J, Malone K, Darling J and Starzyk P (1991) Childhood brain tumor risk in relation to birth characteristics. *J Clin Epidemiol* **44**: 1159–1166

Esteve J, Benhamou E and Raymond L (1994) *Statistical Methods in Cancer Research* Vol. 128. IARC Scientific Publication. IARC: Lyon

Hemminki K and Dong C (2000) Familial relationships in thyroid cancer by histopathological type. *Int J Cancer* **85**: 201–205

Hemminki K and Vaittinen P (1998a) Familial breast cancer in the Family-Cancer Database. *Int J Cancer* **77**: 386–391

Hemminki K and Vaittinen P (1998b) National database of familial cancer in Sweden. *Genet Epidemiol* **15**: 225–236

Hemminki K and Vaittinen P (1999) Familial cancers in a nation-wide family-cancer database: age distribution and prevalence. *Eur J Cancer* **35**: 1109–1111

Hemminki K, Vaittinen P and Kyyrönen P (1998) Age-specific familial risks in common cancers of the offspring. *Int J Cancer* **78**: 172–175

Hemminki K, Kyyrönen P and Vaittinen P (1999) Parental age as a risk factor of childhood leukemia and brain cancer in offspring. *Epidemiol* **10**:271–275

Hemminki K, Li X and Collins V (2000a) Parental cancer as a risk factor for brain tumors (Sweden). *Cancer Causes Control* in press

Hemminki K, Li X and Collins V (2000b) A population-based study of familial central nervous system hemangioblastomas. *Neuroepidemiol* in press

Hemminki K, Li X, Vaittinen P and Dong C (2000c) Cancers in the first-degree relatives of children with brain tumours. *Br J Cancer* **83**: 407–411

Huson S (1998) Neurofibromatosis type 1: historical perspective and introductory overview. In *Neurofibromatosis Type 1*, Upadhyaya M and Cooper D (eds) pp. 1–20. BIOS: Oxford

IARC (1998) *International Incidence of Childhood Cancer* Vol. Vol. II. IARC Sci Publ No. 144. IARC: Lyon

Kaye SA, Robinson LL, Smithson WA, Gunderson P, King FL and Neglia JP (1991) Maternal reproductive history and birth characteristics in childhood acute lymphoblastic leukemia. *Cancer* **68**: 1351–1355

Kinlen L, Dickson M and Stiller C (1995) Childhood leukaemia and non-Hodkin's lymphoma near large rural construction sites, with a comparison with Sellafield nuclear site. *Br J Med* **310**: 763–768

Kleihues P and Cavenee W (2000) *Tumors of the Nervous System*. IARC: Lyon

Lindor N, Greene M and the Mayo Familial Cancer Program (1998) A concise handbook of family cancer syndromes. *J Natl Cancer Inst* **90**: 1039–1071

Linet M, Ries L, Smith M, Tarone R and Devesa S (1999). Cancer surveillance series: recent trends in childhood cancer incidence and mortality in the United States. *J Natl Cancer Inst* **91**: 1051–1058

Little J (1999) *Epidemiology of Childhood Cancer* Vol. 149. IARC Sci Publ. IARC: Lyon

Malmer B, Grönberg H, Bergenheim A, Lenner P and Henriksson R (1999) Familial aggregation of astrocytoma in northern Sweden: an epidemiological cohort study. *Int J Cancer* **81**: 366–370

Narod S, Stiller C and Lenoir G (1991) An estimate of the heritable fraction of childhood cancer. *Br J Cancer* **63**: 993–999

Olsen JH, Boice JD, Seersholm N, Bautz A and Fraumeni JJF (1995) Cancer in the parents of children with cancer. *New Engl J Med* **333**: 1594–1599

Sankila R, Olsen JH, Anderson H, et al (1998) Risk of cancer among offspring of childhood-cancer survivors. *N Engl J Med* **338**: 1339–1344

Savitz D and Ananth C (1994) Birth characteristics of childhood cancer cases, controls, and their siblings. *Pediatr Hematol Oncol* **11**

Sedlacek Z, Kodet R, Poustka A and Goetz P (1998) Database of germline p53 mutations in cancer-prone families. *Nucleic Acids Res* **26**: 214–215

Shaw G, Lavey R, Jackson R and Austin D (1984) Association of childhood leukemia with maternal age, birth order, and parternal occupation. *Am J Epidemiol* **119**: 788–795

Zahm SH and Devessa S (1995) Childhood cancer: overview of incidence trends and environmental carcinogens. *Environ Health Perspect* **103** Suppl 6: 177–184

*© 2001 Cancer Research Campaign*

